# Real-Time Hand Position Sensing Technology Based on Human Body Electrostatics

**DOI:** 10.3390/s18061677

**Published:** 2018-05-23

**Authors:** Kai Tang, Pengfei Li, Chuang Wang, Yifei Wang, Xi Chen

**Affiliations:** State Key Laboratory of Mechatronics Engineering and Control, Beijing Institute of Technology, Beijing 100081, China; tangkai01@bit.edu.cn (K.T.); pfli@bit.edu.cn (P.L.); cunghens@163.com (C.W.); wangyifeibit@163.com (Y.W.)

**Keywords:** real-time hand positioning, non-contact HCI, electrostatics field

## Abstract

Non-contact human-computer interactions (HCI) based on hand gestures have been widely investigated. Here, we present a novel method to locate the real-time position of the hand using the electrostatics of the human body. This method has many advantages, including a delay of less than one millisecond, low cost, and does not require a camera or wearable devices. A formula is first created to sense array signals with five spherical electrodes. Next, a solving algorithm for the real-time measured hand position is introduced and solving equations for three-dimensional coordinates of hand position are obtained. A non-contact real-time hand position sensing system was established to perform verification experiments, and the principle error of the algorithm and the systematic noise were also analyzed. The results show that this novel technology can determine the dynamic parameters of hand movements with good robustness to meet the requirements of complicated HCI.

## 1. Introduction

Natural, harmonious, and highly efficient human-computer interactions (HCI) have become a trend in the field of human-computer interaction research. Human hand motion-based human-computer interaction is one of the most important methods used in human-computer interactions [1]. Generally, hand motion sensing systems can be divided into: data glove-based capturing, attached force-based capturing, surface electromyography (SEMG)-based capturing, optical markers-based capturing, and vision-based capturing [2], which are mainly divided into wearable hand motion sensing and vision-based hand motion sensing methods [1,3]. The two methods have their own characteristics. Wearable gesture recognition has high recognition accuracy that can capture the movement details of the hand. Vision-based gesture recognition obtains gesture information in a non-contact manner and can be applied to a wider range of fields. However, wearable and vision-based gesture recognition methods have disadvantages, including poor user experience and vulnerability to environmental factors such as illumination [1,4]. Wearable electromyography (EMG) signal control systems require electrodes to be stuck onto the forearm or wrist, which requires skin cleanliness and electrode performance with certain use restrictions [5]. Wearable data gloves are not only inconvenient to use, and shifts in the data glove can easily cause errors in the system [1,3]. The vision-based gesture interaction method, in addition to certain requirements for light and background, are of relatively high computational complexity, perform poorly in real-time, and have other shortcomings in terms of target recognition and processing; for example, very complex feature representations and inaccurate feature extractions caused by incorrect human segmentation [6]. For an accuracy of 90% or higher, the average processing time is longer than 30 ms [7]. There is a gesture recognition method based on terahertz radar and the Doppler signal that avoids the disadvantages of the above two methods. However, the terahertz radar is large, expensive and not suitable for development [8].

Because the human body is electrostatically charged in certain environments for a variety of reasons, when the hand moves, the electrostatic charge of the hand will disturb the space’s electric field. The disturbed electric field will lead to an electrostatic induction on the electrode in space, thus hand movement can be verified by detecting the electrostatic induction signal on the electrode. Detecting the hand position using the human body’s own electrostatic field is a method for recognizing human hand movements under non-contact conditions that is not influenced by environmental factors such as light [9]. Therefore, the study of real-time hand position sensing technology based on human body electrostatics can obtain hand movement trajectories via electrostatics detection using a non-contact method, avoiding the disadvantages of being visually sensitive to light and complicated by background interference, preventing a poor user experience. This research will lay the theoretical and practical foundation for new non-contact human–computer interaction methods, which will be of practical significance.

In the field of human body electrostatic detection, the Center for Physical Electronics and Quantum Technology (CPEQT) at the University of Sussex in the U.K. conducted an in-depth study of the electrostatic signal detection of the human heartbeat that was reported in *Nature* [10,11,12]. In the field of human body movement detection and human–computer interaction, Takiguchi introduced and applied a non-contact electrostatic detection system to detect the moving human body [13,14]. Kurita detected human foot movements with a non-contact electrostatics detection method and applied it to throwing movement analysis [15,16,17,18,19]. Four detection electrodes were used to measure the movement of the hand in eight directions and applied to human–computer interactions. In 2015, we conducted research on sensing the direction and velocity parameters of human hand movements with an electrostatic electrode array. Theoretical and experimental studies confirmed that the direction and velocity parameters of the hand movement can be obtained with the human body’s electrostatic information and can be applied to simple interactive games [9].

In our previous works, only the direction and velocity of human hand movements were obtained using the electrostatic detection method, and the spatial position of the operator’s hand was not obtained in real time, which restrained the application of this technology. With the rapid development of human–computer interaction technology, increasing application demands for complex operations in three-dimensional (3D) space, such as interactive operation in the Virtual Reality/Augmented Reality (VR/AR) environment and 3D modeling, the precise positioning of the operator’s hand position is required. To this end, we further developed a real-time hand position algorithm based on the electrostatic information from the human body, designed a new electrostatic electrode array structure and a new solving method to solve the position of the charge source, achieving real-time human hand positioning.

## 2. Sensing Principle and Positioning Algorithm

In this paper, five-spherical-electrode sensing array sensing signals are established, and the method used to calculate the position of the hand in real time is studied. The solution of the 3D coordinates of the hand position is obtained, and the movement of the hand is obtained using the constrained scanning positioning method. By locating the charge source, the human hand real-time position can be obtained. The algorithms are specified in Section 3.

The induction signal expression of the five-spherical-electrode detection array and the principle of “constraint scanning positioning method” are mainly analyzed in this section.

### 2.1. Electrostatic Field Sensing Principle

As a charged body, with the hand being the tip, it can produce a disturbance on the electric field of surrounding space by using hand motion. Based on the theories of electrodynamics, as the hand movement changes the electric fields in space, the induced charge on the surface of the conductor redistributes, leading to the generation of induced current on the conductor.

**Figure 1:**
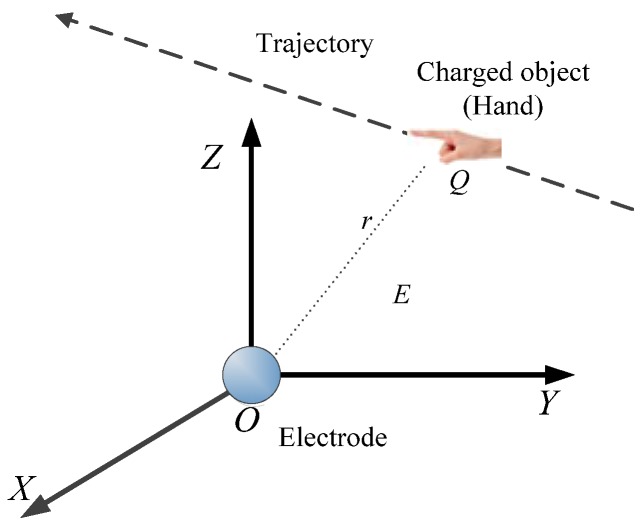
Schematic of the spherical electrode.

In this study, the induction signals of spherical electrodes were quantitatively analyzed. Figure 1 shows the positional relationship between the spherical electrode and the hand target *T*. The spherical electrode is at the origin of the coordinate, the hand charge quantity is *Q*, the hand moves along a certain trajectory, and the center of the hand has a distance *r* from the center of the spherical electrode.

According to the previous research results, when the human hand moves, the induced current signal of the spherical electrode can be expressed as [7]:

(1) $$i=\frac{dQ_{E}}{\mathrm{dt}}=\frac{d\left(-\frac{R_{0}}{r}Q\right)}{dt}=\frac{QR_{0}}{r^{2}}\frac{dr}{dt}$$

*Q*_0_ is the target (hand) charge quantity. *R*_0_ is the spherical electrode radius. *r* is the distance from the hand to the spherical electrode.

**Figure 2:**
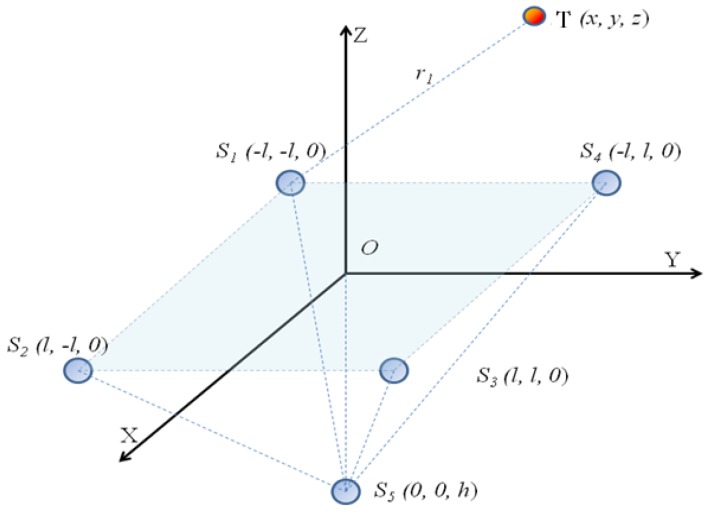
Planar electrode layout.

The real-time position of the 3D space of the hand movement was detected with a five-spherical- electrode sensing array, and a Cartesian coordinate system model of the detection array was established, as shown in Figure 2.

Coordinates of the charged target *T* are (*x*, *y*, *z*), with a charge quantity of *Q*_0_. Electrodes *S*_1_*, S*_2_*, S*_3_, and *S*_4_ are located at the four vertices of the square centered on the origin *O* and with 2*l* being the side length on the *XOY* plane. The coordinates of the four vertices are *S*_1_ (*−l*, *−l*, *0*), *S*_2_ (*l*, *−l*, *0*), *S*_3_ (*l*, *l*, *0*), and *S*_4_ (*−l*, *l*, *0*). The electrode *S*_5_ was set on the *z*-axis with the coordinates (*0*, *0*, *−h*).

Let *S* be the effective sensing area of the spherical electrode and *R*_0_ the spherical electrode radius. According to the Gaussian theorem, the induced charge of the electrode *i*, *i* = (1, 2, 3, 4, 5) under the electric field of the charge source T is:

(2) $$\left\{ \begin{array}{c} i_{1}=\frac{Q_{0}S}{4\pi }\left\{\begin{array}{c} \frac{dz}{dt}\cdot \frac{1}{{\left(x+l\right)}^{2}+{\left(y+l\right)}^{2}+z^{2}}-2[\frac{x+l}{{\sqrt{{\left(x+l\right)}^{2}+{\left(y+l\right)}^{2}+z^{2}}}^{2}}\cdot \frac{dx}{dt}+\frac{y+l}{\sqrt{{\left(x+l\right)}^{2}+{\left(y+l\right)}^{2}+z^{2}}}\cdot \frac{dy}{dt}+\frac{z}{\sqrt{{\left(x+l\right)}^{2}+{\left(y+l\right)}^{2}+z^{2}}}\cdot \frac{dz}{dt}]\\ \cdot \frac{z}{{\left[{\left(x+l\right)}^{2}+{\left(y+l\right)}^{2}+z^{2}\right]}^{2}} \end{array}\right\}\\ i_{2}=\frac{Q_{0}S}{4\pi }\left\{\begin{array}{c} \frac{dz}{dt}\cdot \frac{1}{{\left(x-l\right)}^{2}+{\left(y+l\right)}^{2}+z^{2}}-2[\frac{x-l}{{\sqrt{{\left(x-l\right)}^{2}+{\left(y+l\right)}^{2}+z^{2}}}^{2}}\cdot \frac{dx}{dt}+\frac{y+l}{\sqrt{{\left(x-l\right)}^{2}+{\left(y+l\right)}^{2}+z^{2}}}\cdot \frac{dy}{dt}+\frac{z}{\sqrt{{\left(x-l\right)}^{2}+{\left(y+l\right)}^{2}+z^{2}}}\cdot \frac{dz}{dt}]\\ \cdot \frac{z}{{\left[{\left(x-l\right)}^{2}+{\left(y+l\right)}^{2}+z^{2}\right]}^{2}} \end{array}\right\}\\ i_{3}=\frac{Q_{0}S}{4\pi }\left\{\begin{array}{c} \frac{dz}{dt}\cdot \frac{1}{{\left(x-l\right)}^{2}+{\left(y-l\right)}^{2}+z^{2}}-2[\frac{x-l}{{\sqrt{{\left(x-l\right)}^{2}+{\left(y-l\right)}^{2}+z^{2}}}^{2}}\cdot \frac{dx}{dt}+\frac{y-l}{\sqrt{{\left(x-l\right)}^{2}+{\left(y-l\right)}^{2}+z^{2}}}\cdot \frac{dy}{dt}+\frac{z}{\sqrt{{\left(x-l\right)}^{2}+{\left(y-l\right)}^{2}+z^{2}}}\cdot \frac{dz}{dt}]\\ \cdot \frac{z}{{\left[{\left(x-l\right)}^{2}+{\left(y-l\right)}^{2}+z^{2}\right]}^{2}} \end{array}\right\}\\ i_{4}=\frac{Q_{0}S}{4\pi }\left\{\begin{array}{c} \frac{dz}{dt}\cdot \frac{1}{{\left(x+l\right)}^{2}+{\left(y-l\right)}^{2}+z^{2}}-2[\frac{x+l}{{\sqrt{{\left(x+l\right)}^{2}+{\left(y-l\right)}^{2}+z^{2}}}^{2}}\cdot \frac{dx}{dt}+\frac{y-l}{\sqrt{{\left(x+l\right)}^{2}+{\left(y-l\right)}^{2}+z^{2}}}\cdot \frac{dy}{dt}+\frac{z}{\sqrt{{\left(x+l\right)}^{2}+{\left(y-l\right)}^{2}+z^{2}}}\cdot \frac{dz}{dt}]\\ \cdot \frac{z}{{\left[{\left(x+l\right)}^{2}+{\left(y-l\right)}^{2}+z^{2}\right]}^{2}} \end{array}\right\}\\ i_{5}=\frac{Q_{0}S}{4\pi }\left\{\begin{array}{c} \frac{dz}{dt}\cdot \frac{1}{x^{2}+y^{2}+{\left(z+h\right)}^{2}}-2[\frac{x}{x^{2}+y^{2}+{\left(z+h\right)}^{2}}\cdot \frac{dx}{dt}+\frac{y}{\sqrt{x^{2}+y^{2}+{\left(z+h\right)}^{2}}}\cdot \frac{dy}{dt}+\frac{z+h}{\sqrt{x^{2}+y^{2}+{\left(z+h\right)}^{2}}}\cdot \frac{dz}{dt}]\\ \cdot \frac{z}{{\left[x^{2}+y^{2}+{\left(z+h\right)}^{2}\right]}^{2}} \end{array}\right\} \end{array}\right.$$

Equation (2) is the induced current output of each element of the five-spherical-electrode detection array.

### 2.2. Hand Position Solving Algorithm Based on Human Body Electrostatic Forces

By integrating the induced current of each element of the five-spherical-electrode detection array, the induced charge quantity of all electrodes can be obtained. Calculate the induced charge ratio $\frac{q_{2}}{q_{1}}$,$\;\frac{q_{3}}{q_{2}}$, $\frac{q_{5}}{q_{1}}$ of electrodes 1 to 5. The charge of the charge source is eliminated to obtain an equation containing only the position parameters. The geometric constraint is applied to constrain the charge source position to a circle. Finally, scan the position of the charge source on the circle, and determine the 3D coordinates of the charge source by using the charge quantity of electrode 5 on the *z*-axis, which is called the constraint scanning positioning method.

Step 1 Current integration to obtain the charge quantity

Integrating the detection from Equation (2), *Q*_1_–*Q*_5_ can be obtained:

(3) $$Q_{i}=\int_{0}^{t}i_{i}dt\;(i=1,2,3,4,5)$$

Step 2: Ratio elimination

Calculate the ratio of the induced charge quantity of each electrode to eliminate the charge quantity of the charge source, and we can obtain:

(4) $$\frac{Q_{2}}{Q_{1}}={\left(\frac{r_{1}}{r_{2}}\right)}^{2}=k_{1},\;\frac{Q_{3}}{Q_{2}}={\left(\frac{r_{2}}{r_{3}}\right)}^{2}=k_{2},\;\frac{Q_{4}}{Q_{3}}={\left(\frac{r_{3}}{r_{4}}\right)}^{2}=k_{3},\;\frac{Q_{5}}{Q_{1}}={\left(\frac{r_{1}}{r_{52}}\right)}^{2}=k_{4}$$

Step 3: Spherical constraint

Calculate the square root of ${\left(\frac{r_{1}}{r_{2}}\right)}^{2}=k_{1}$ in Equation (4) to obtain:

(5) $$\frac{r_{1}}{r_{2}}=\sqrt{k_{1}}$$

where *r*_1_ is the distance between the charge source *T* and electrode *S*_1_, and *r*_2_ is the distance between charge source *T* and *S*_2_. According to the sphere definition, “the trajectory of all the points with constant ratio of distances to two fixed points” [20], all the points satisfying Equation (5) can form a sphere, so the charge source *T* can be constrained on this sphere. The center and radius of the sphere are calculated below.

**Figure 3:**
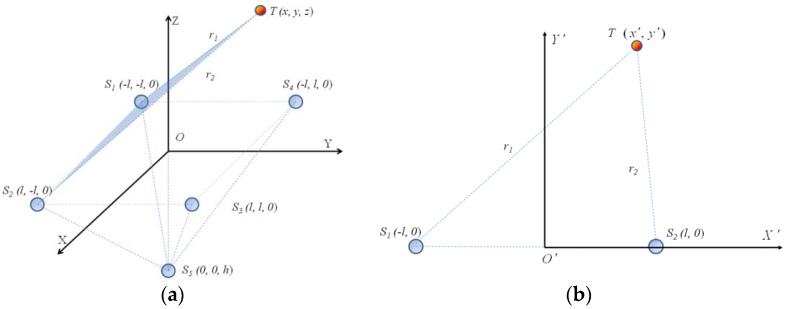
Schematic of coordinate transformation: (**a**) three-dimensional (3D) coordinates and (**b**) two-dimensional (2D) coordinates.

To more easily determine the spherical center and radius, coordinate system transformation is implemented first, as shown in Figure 3. Point *T* and the electrodes *S*_1_ and *S*_2_ are transformed from the 3D coordinate system to the plane coordinate system composed of *T*, *S*_1_, and *S*_2_. Since the *S*_1_ and *S*_2_ points are on the YOZ plane, the coordinate transformation does not change the distance between two points.

Suppose point *T* to be (*x’*, *y’*), *S*_1_ (*−l*, *0*), *S*_2_ (*l*, *0*) in the plane coordinate system *X’O’Y’*. If *k*_1_ *=* 1, point *T* is on the bisector plane of line *S*_1_ *S*_2_. If *k*_1_ ≠ 1, then the square of the distance ratio from point *T* to *S*_1_, *S*_2_ can be written as:

(6) $$\frac{{\left(x^{\prime }+l\right)}^{2}+y{}^{\prime }{}^{2}}{{\left(x^{\prime }-l\right)}^{2}+y{}^{\prime }{}^{2}}=k_{1}$$

Substituting Equation (6) into the equation of a circle:

(7) $${\left(x^{\prime }+\frac{1+k_{1}}{1-k_{1}}l\right)}^{2}+y{}^{\prime }{}^{2}=[{\left(\frac{1+k_{1}}{1-k_{1}}\right)}^{2}-1]l^{2}$$

That is, on the plane X’O’Y’, point *T* is on the circle with center A’(−$\frac{1+k_{1}}{1-k_{1}}l,\;0$), and radius $r_{1}{}^{\prime }=\sqrt{[{\left(\frac{1+k_{1}}{1-k_{1}}\right)}^{2}-1]l^{2}}$. The center of circle A’ is collinear with *S*_1_*, S*_2_.

When transforming to the *XYZ* coordinate system, because the center position and the radius do not change when the coordinate transformation is performed and the center of circle *A’* is collinear with *S*_1_, *S*_2_, the center of circle is in the *XOY* plane in *XYZ* coordinate system. Therefore, in *XYZ* coordinate system, the distance between points *T* and *A* (−$\frac{1\;+\;k_{1}}{1\;-\;k_{1}}l,\;-l,0$) is $\sqrt{[{\left(\frac{1\;+\;k_{1}}{1\;-\;k_{1}}\right)}^{2}-1]l^{2}}$. All points *T* on the sphere with *A* as the center and $\sqrt{[{\left(\frac{1\;+\;k_{1}}{1\;-\;k_{1}}\right)}^{2}-1]l^{2}}$ as the radius satisfy Equation (5).

Similarly, in the plane *TS*_2_*S*_3_, the distance between point *T* and point *B*(−$\frac{1\;+\;k_{2}}{1\;-\;k_{2}}l,\;0$) is $r_{2}{}^{\prime }=\sqrt{[{\left(\frac{1\;+\;k_{2}}{1\;-\;k_{2}}\right)}^{2}-1]l^{2}}$. When transforming to *XYZ* space, the distance between point *T* and *B* (*l*, −$\frac{1\;+\;k_{2}}{1\;-\;k_{2}}l,\;0$) is $\sqrt{[{\left(\frac{1\;+\;k_{2}}{1\;-\;k_{2}}\right)}^{2}-1]l^{2}}$. Then point *T* can be constrained to the sphere with *B* as the center and $\sqrt{[{\left(\frac{1\;+\;k_{2}}{1-k_{2}}\right)}^{2}-1]l^{2}}$ as the radius.

In the plane *TS*_3_*S*_4_, the distance between *T* and *C*’(−$\frac{1\;+\;k_{3}}{1\;-\;k_{3}}l,\;0$) is $r_{3}{}^{\prime }=\sqrt{[{\left(\frac{1\;+\;k_{3}}{1\;-\;k_{3}}\right)}^{2}-1]l^{2}}$. When transforming to *XYZ* space, the distance between point *T* and *C* ($\frac{1\;+\;k_{3}}{1\;-\;k_{3}}l,l,\;0$) is $\sqrt{[{\left(\frac{1\;+\;k_{3}}{1\;-\;k_{3}}\right)}^{2}-1]l^{2}}$. Then point *T* can be constrained to the sphere with *C* as the center and $\sqrt{[{\left(\frac{1\;+\;k_{3}}{1\;-\;k_{3}}\right)}^{2}-1]l^{2}}$ as the radius.

Step 4: Circular constraint

Since *S*_1_, *S*_2_, *S*_3_, and *S*_4_ are coplanar, it is easy to prove that *A* ($-\frac{1\;+\;k_{1}}{1\;-\;k_{1}}l,\;-l,0$), *B*(−$\frac{1\;+\;k_{2}}{1\;-\;k_{2}}l,\;0$), and *C* ($\frac{1\;+\;k_{3}}{1\;-\;k_{3}}l,l,\;0$) are collinear. So, the three spherical surfaces with *A*, *B*, and *C* as centers obtained in the spherical constraint step will intersect on a circle, so one of the spheres is redundant. Taking the two spherical surfaces with A and B as centers as an example, implement the circular constraint.

**Figure 4:**
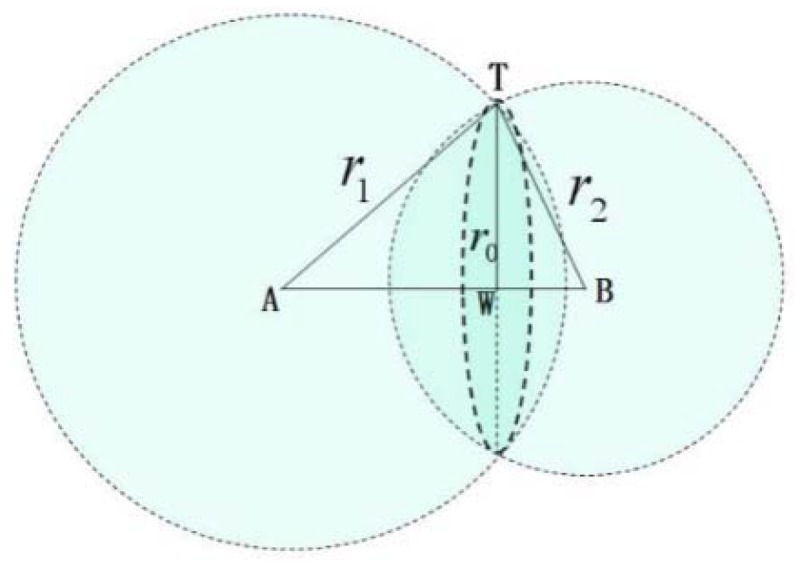
Schematic of circular constraint diagram.

As shown in Figure 4, through the above-mentioned two points, *A* and *B*, point *T* can be constrained to two spherical surfaces that intersect to a circle, meaning point *T* is further constrained to a circle with radius of *r*_0_ and center *W*. Since the center *W* is on the line AB, the center *W* is on the *XOY* plane, and the coordinates of *W* are (x_0_, y_0_, 0). The specific values of x_0_, y_0_ and r_0_ can be obtained from the coordinates of points *A* and *B*, *r*_1_ and *r*_2_, through the geometric relations.

From a mathematical point of view, the two spherical surfaces with *A* and *B* as centers can constrain the charge sources to a circle, but during the actual use of the system, due to systematic error, the positions of *A* and *B* may have some deviations. To improve the accuracy of the circular constraint, it can be further improved by straight line fitting of *A*, *B*, and *C*.

Through *A*, *B*, *C* straight line fitting:

(8) y=ax+b

According to the positions of *A*, *B*, *C* and *r*_1_, *r*_2_, *r*_3_, we can determine that point *T* is located on the circle with *r*_0_ as the radius and center *W* (x_0_, y_0_, 0). The line in Equation (8) is vertical to the plane where the circle is located and through the center *W* of the circle.

Step 5: Angle Scanning

Represent the charge source *T* with the polar coordinates on the circle determined in Step 4. Set the angle between the TW connection line and plane *XOY* as θ, with a scan value of θ, so that when point *T* moves along the circle, the position of point *T* can be obtained with Equation (4).

**Figure 5:**
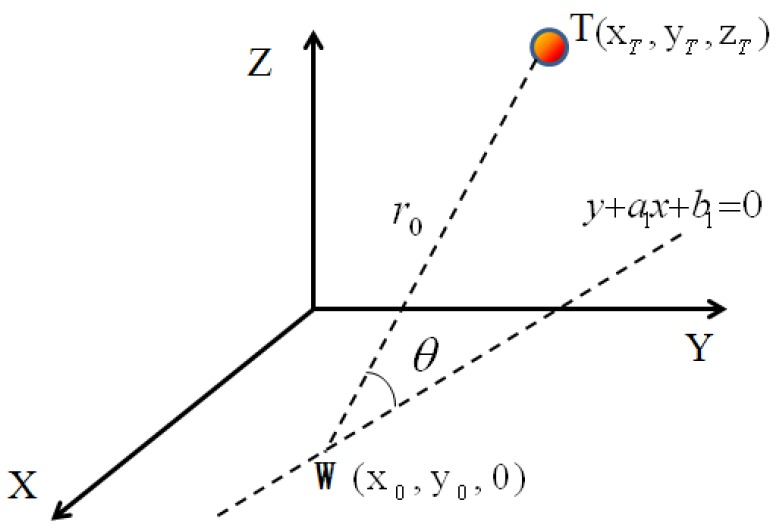
Schematic of angle scanning.

As shown in Figure 5, set 0 < θ < π, as the hand is always in the *z*-axis positive direction side in human–computer interactions. The projection of TW on the plane *XOY* is a straight line y = a_1_x + b_1_, a_1_ = $-\frac{1}{a}$, where *a* is the slope of the straight line in Equation (8). Then the coordinates of point *T* are converted to polar coordinates:

(9) $$\{\begin{array}{c} x_{T}=r_{0}cos\theta \frac{1}{\sqrt{a_{1}{}^{2}+1}}+x_{0}\\ y_{T}=r_{0}cos\theta \frac{1a_{1}}{\sqrt{a_{1}{}^{2}+1}}+y_{0}\\ z_{T}=r_{0}sin\theta \end{array}$$

Scan *θ* in the range of (0, π) according to a step size, so that:

(10) $$\frac{{\left(x_{T}-x_{1}\right)}^{2}+{\left(y_{T}-y_{1}\right)}^{2}{\left(z_{T}-z_{1}\right)}^{2}}{{\left(x_{T}-x_{5}\right)}^{2}+{\left(y_{T}-y_{5}\right)}^{2}{\left(z_{T}-z_{5}\right)}^{2}}=\frac{Q_{5}}{Q_{1}}$$

where $\left(x_{1},y_{1},z_{1}\right)$ and $\left(x_{5},y_{5},z_{5}\right)$ are the positions of electrodes 1 and 5, respectively, and $\left(x_{1},y_{1},z_{1}\right)$ is (*−l*, *−l*, *0*) and $\left(x_{5},y_{5},z_{5}\right)$ is (*0*, *0*, *h*) in the system. At this time, $\left(x_{T},y_{T},z_{T}\right)$ in Equation (9) is the actual coordinates of the hand.

The real-time position of the hand can be obtained by calculating the spatial coordinates of the charge source with a small amount of computation and high accuracy.

The calculated position of the hand is affected by the noise of the system, which will cause some errors. Since the hand motion is a continuous trajectory, the Kalman filtering method can be used to reduce the hand position resolution error and improve the system accuracy. When the system sampling rate is 1k, the calculated distance between the adjacent hand positions is on the millimeter scale, and the distance is very close. This can be approximated as a rectilinear motion between the adjacent hand positions, so we use a rectilinear motion model and select velocity covariance and acceleration covariance to do the Kalman filtering.

### 2.3. Design and Experiment of Non-Contact Interactive System Based on Human Body Electrostatic Forces

**Figure 6:**
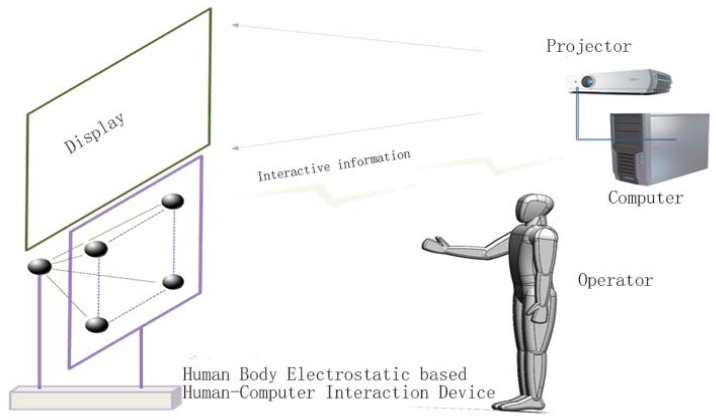
Schematic of human-computer interaction (HCI) system based on the electrostatics of the human body.

Using the electrostatic array layout and signal processing, the human–computer interactive system based on the electrostatics of the human body can obtain human hand movement parameter information, such as angle, direction, speed, or real-time 3D positions, obtain the hand movement trajectory, judge the operation intentions of operators, control and operate the software running on the computer, and complete the human-machine interactive function. Figure 6 shows the application schematic diagram of our human-machine interactive system based on the electrostatics of the human body. With the display devices (display screen and projector) and computer, the system can allow operators to perform the gesture human–computer interactive operations with various 3D software, such as virtual assembly, virtual experiment, and 3D modeling software, under a variety of lighting and complex backgrounds without wearing any sensing equipment.

**Figure 7:**
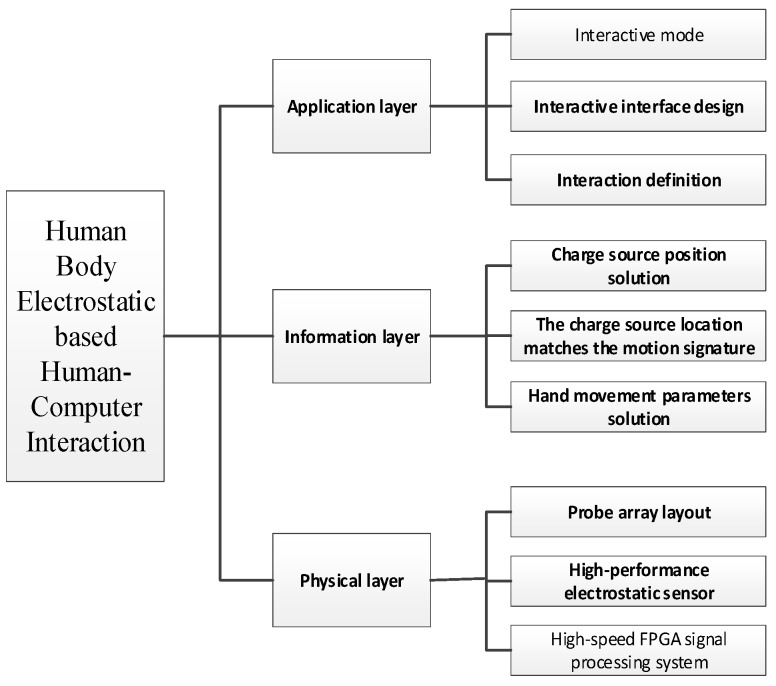
Architecture diagram depicting the overall scheme.

We designed a top-level human–computer interaction system based on the electrostatics of the human body, built the hierarchical architecture, and divided the entire human–computer interaction system into physical, information, and application layers. The physical layer is the physical basis of the whole system, including the sensing circuits and signal processing circuit hardware. The information layer processes the signals from the physical layer, and mainly performs model and algorithm research, including solving for the position information of the charge sources according to the source azimuth solving model of electric fields. The information layer also performs mode recognition and matching calculations for gesture information patterns, and finally outputs the standard human hand movement identification information. The application layer mainly completes research to output data from the information layer, constructs the human-machine interactive mode through the definition of human gestures, and designs the human-machine interactive interface to complete the HCI function based on electrostatic detection. The overall scheme is shown in Figure 7.

**Figure 8:**
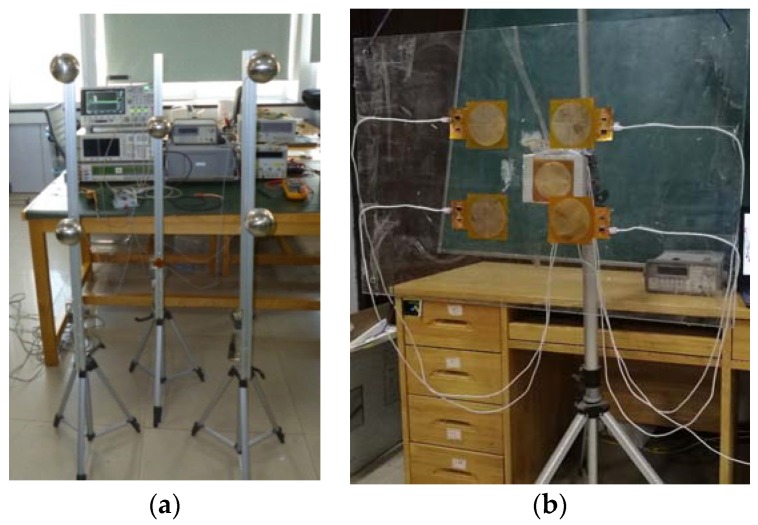
Photograph of our experimental equipment. (**a**) spherical electrode device and (**b**) round planar electrode device.

We built a real-time position measurement system for non-contact HCI based on the electrostatic signals, as shown in Figure 8a. The system mainly consists of five-spherical-electrode, electrostatic sensing circuits, and data acquisition processing units. The five-spherical-electrode are arranged in the positions shown in Figure 8a, and the vertical plane is defined as the *XOY* plane. The four spherical electrodes are S_1_–S_5_, arranged at the four corners of the square with a side length of 0.3 m on the *XOY* plane. The square center is the origin of the coordinates, and the fifth spherical electrode is S_5_. As the center electrode, S_5_ is arranged on the *z*-axis 0.2 m away from the *XOY* platform. We used a round planar electrode instead of a spherical electrode to make the same experimental device. As shown in Figure 8b, this device has the same performance parameters as a spherical electrode device and has a more compact structure. In actual use, the electrodes can be made of transparent conductive materials such as Indium Tin Oxide (ITO) and integrated with the display screen to accommodate more application scenarios.

## 3. Results

### 3.1. Hand Real-Time Position Acquisition

A real-time position sensing system for non-contact HCI based on human body electrostatics was built and verified by experiments. The operator stood 1.5 m away from the electrode plane and moved one hand in the air. The trajectory of the hand movement was calculated using the induced current data measured by the electrode.

**Figure 9:**
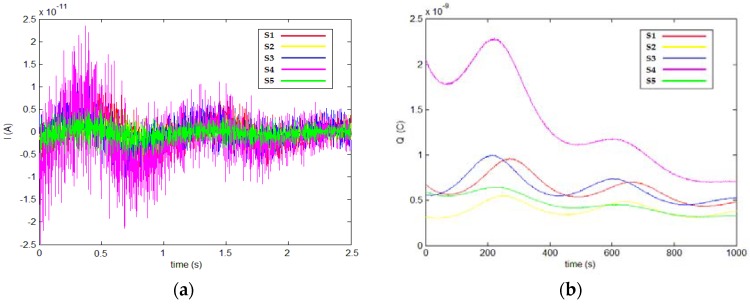
(**a**) Induced current curve and (**b**) induced charge curve after processing.

**Figure 10:**
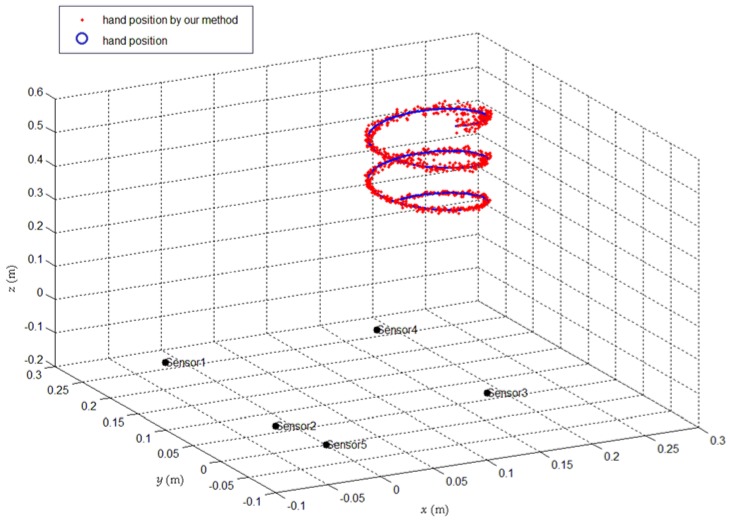
Hand position and trajectory curve obtained by constrained scanning positioning method.

The results are shown in Figure 9 and Figure 10. The operator’s spiral gesture trajectory was detected, and the induced current detection curve was obtained as shown in Figure 9a. The obtained induced charge curve after processing is shown in Figure 9b.

We calculated the position points of the hand movement trajectory, and the calculated hand position is indicated by the red dots in Figure 10. The smooth hand movement trajectory obtained through Kalman filtering is indicated by a blue line and the time delay was less than one millisecond.

### 3.2. System Accuracy Analysis and Verification

We used Leap Motion as a standard detection system to verify the accuracy of the electrostatic detection system. We placed the Leap Motion in the front of the spherical electrode plane so that both the Leap Motion and the five-electrode sensing array can sense hand motion. When the hand moves, Leap Motion acquires the trajectory of the hand, and its positioning accuracy is better than that of the millimeter scale. At the same time, our system also acquires hand movement trajectories. We adjust the coordinate system and sampling rate of the two measurement systems so that the two systems have the same measurement reference. The distance between the corresponding measurement data of the two systems is calculated as the measurement error of our system. Through this method, the accuracy of our system is verified.

**Figure 11:**
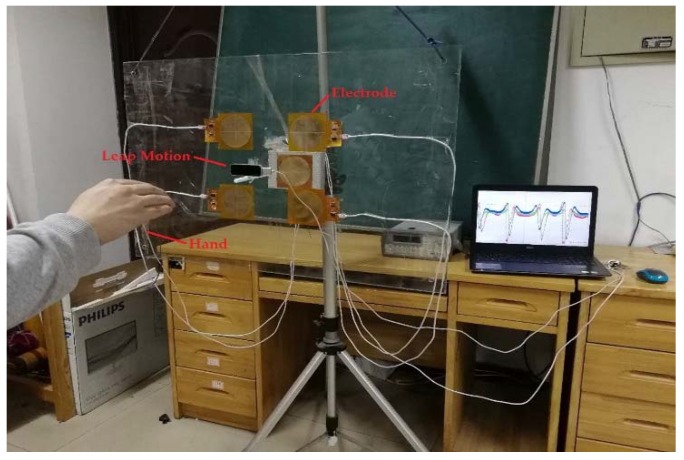
Experimental scene.

The experimental scenario is shown in Figure 11. A person at a distance of 1.5 m from the electrode plane drew circles in the air, and the hand movement trajectory was solved using the induced current data measured by the electrode.

**Figure 12:**
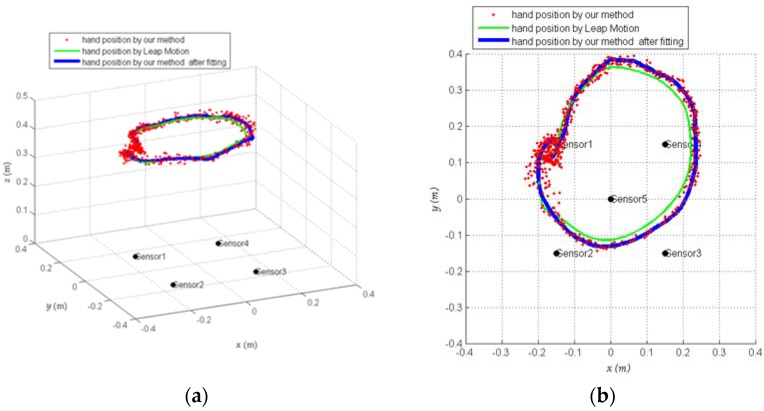
Measured results. (**a**) Side view and (**b**) top view.

The solving result is shown in Figure 12, in which the red indicates the hand position of solving, and the blue indicates the hand movement trajectory after fitting, and the green indicates the hand movement trajectory from Leap Motion. From Figure 12, the hand positions obtained after measurement and calculation were generally distributed around a circular trajectory, so we fit the calculated hand positions by using the Kalman filtering method, and the fitted movement trajectory can better follow the circular movement trajectory of the hand.

After testing by eight experimenters with different heights, genders and clothing, the detection range of the system is 0.1 m–2.3 m, which can respond to the movement of the experimenter’s hand 1–2 cm. However, Leap Motion’s detection range is 0.025 to 0.6 m. Therefore, we tested the accuracy of the electrostatic detection system by move the placement of the Leap Motion to detect the accuracy within the detection range of 0.1 to 2.3 m.

**Figure 13:**
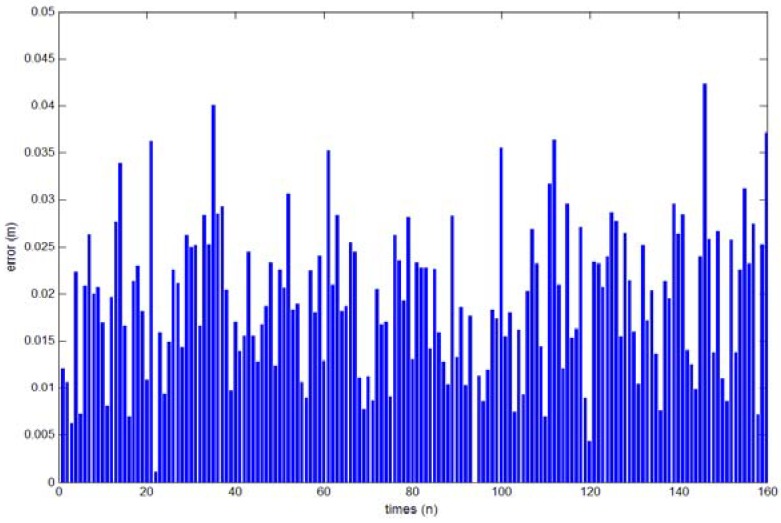
Position error result.

We determined the accuracy of the system by using the experimental data of 160 groups of hand circles collected by eight experimenters as shown in Figure 13.

We analyzed the hand position errors for the measuring results, and the results are shown in Figure 13. In Figure 13, the horizontal axis is *n*, and the vertical axis indicates the solved hand position errors with units of *m*. The maximum error of 0.043 m is seen in Figure 13, which can meet the needs of human–computer interaction systems in 3D space such as play games or handwriting.

## 4. Discussion

According to the experimental results, an accurate hand movement trajectory curve was obtained using our real-time position sensing system based on the electrostatic hand signals. However, the obtained hand position had a certain error, distributed around the hand’s actual position. The real-time hand position sensing system model was established by using MATLAB software. The hand position calculation under an ideal situation and the hand position calculation under the condition of adding the measurement error were completed. The precision of the hand locus resolution was analyzed using different system measurement errors.

### 4.1. Situation with Measurement Error of Zero

The quantity of charge source was set at 10^−9^ C in simulation, with an electrode spacing of 0.3 m, and electrode radius of 0.025 m, so that the charge source *T* can move at a velocity of one m/s following the set path. The induced current and induced charge quantity of each electrode were calculated, and the 3D coordinates of the charge source were solved in real time with the constrained scan positioning method. The positioning error was obtained by comparing the actual coordinates with the charge source.

**Figure 14:**
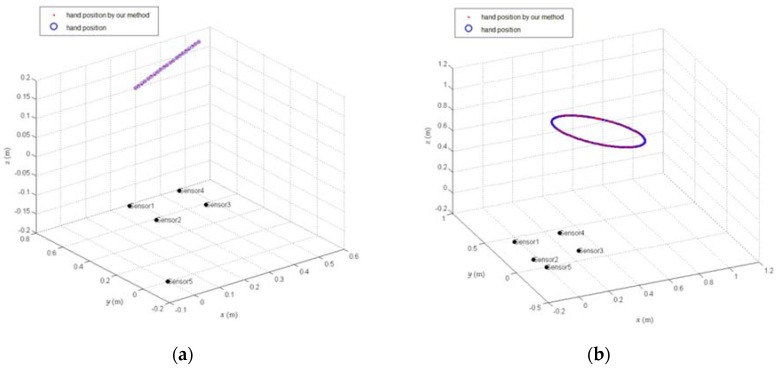
Charge source real-time position solution results for (**a**) a linear trajectory and (**b**) a circular trajectory.

The simulation results are shown in Figure 14, in which green represents the actual position of charge source, and the red represents the calculated position of the charge source. The solved position and the actual position are strictly consistent.

From the above two simulations, we concluded that, in an ideal case without noise, the real-time solution accuracy of the charge source is very high, meaning the algorithm has no principle error.

### 4.2. Adding Measurement Error

Since the system uses a high-gain amplifier circuit to detect the weak current signal, the thermal noise and shot noise are the most important system noise, and are considered the main source of measurement error. The thermal noise and shot noise of the measurement circuit were simulated by adding a Gaussian white noise analog to the simulation, and the positioning error caused by the measurement error of the system was analyzed.

**Figure 15:**
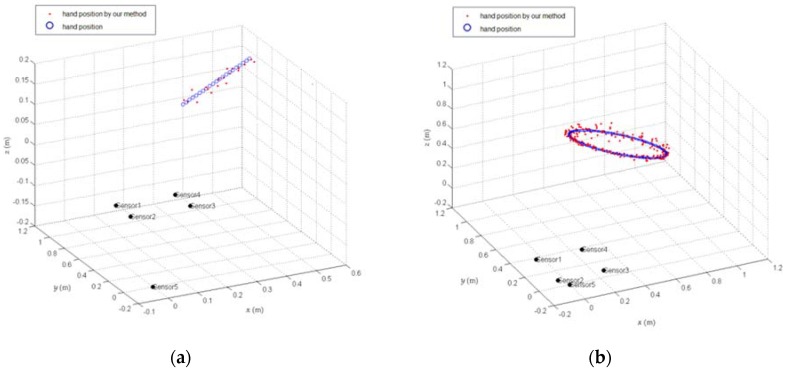
Position solution results by adding 10% noise into signal for (**a**) a linear trajectory and (**b**) a circular trajectory.

Figure 15 shows the solving result of the position of the charge source by adding 10% noise into the signal, in which blue represents the actual position of charge source and red denotes the position of charge source calculated by the induced charges of the five electrodes.

**Figure 16:**
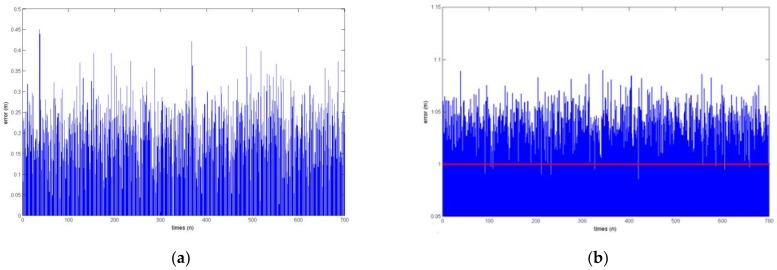
(**a**) Position and (**b**) velocity deviation results with 10% noise in the signal.

Figure 16 shows the position error and velocity distribution of the charge source with 10% noise. Figure 16a shows the result of the position error analysis of the charge source, with an abscissa of *n* and an ordinate of the calculated position error of the charge source in *m* units. The maximum error was 0.045 m. Figure 16b shows a comparison between the actual velocity and the calculated charge source moving velocity, with abscissa of *n*, and ordinate of the velocity value in m/s. The red line represents the actual velocity of the charge source, which is 1 m/s, and the blue line represents the calculated charge source moving velocity, with a maximum of 1.08 m/s, and an error of less than 8%.

The experimental results show that when positioning the human hand with the human body electrostatic signal, noise has some influence on solving the hand position and velocity, but the maximum distance error was less than 0.045 m with 10% noise, and the velocity error did not exceed 8%.

Because the position error caused by the system measurement error is a random error and a sudden change will not occur in the hand position during movement; the hand moves in a continuous trajectory. Therefore, the influence of a random error can be reduced by center fitting of adjacent position points or by Kalman filter improving the positioning accuracy.

## 5. Conclusions

The method we introduced to solve the real-time hand position was able to obtain the position of the hand with high accuracy by acquiring the hand’s electrostatic signal. There is no principle error in the method. The hand real-time position sensing system has some positioning error due to the systematic measurement error. The maximum distance error was less than 0.045 m and the velocity error was not more than 8% when the noise is 10%. This error can be further reduced by multi-point fitting.

After the subject’s movement trajectory is further identified, the function of mouse-like interactive functions such as selecting, dragging, opening, closing through the combination of hand positions and hand motion trajectories, and handwriting input can also be achieved. We have already started this research and will present it in a later paper.

Therefore, the real-time hand movement position tracking and calculation by measuring the human hand movement electrostatic signal can better determine the hand movement trajectory, meeting the higher accuracy, more complex hand movement trajectory tracking, and human–computer interaction demands.

## 6. Patents

Xi Chen; Kai Tang; Pengfei Li; Wei Wang. Motion charge source real-time location sensing method. 201610516862.9, 2016.11.16.

Pengfei Li; Xi Chen; Kai Tang; Chuang Wang. Motion charge source movement speed and direction sensing method. 201610516790.8,2016.10.26.
